# Dengue virus and lipid metabolism: unravelling the interplay for future therapeutic approaches

**DOI:** 10.1080/22221751.2025.2477647

**Published:** 2025-03-10

**Authors:** Ying Xie, Li Jiao, Qiangming Sun

**Affiliations:** a Institute of Medical Biology, Chinese Academy of Medical Sciences & Peking Union Medical College, Kunming, People's Republic of China; b Kunming Medical University, Kunming, People’s Republic of China; c State Key Laboratory of Respiratory Health and Multimorbidity, Beijing, People’s Republic of China; d Yunnan Key Laboratory of Vaccine Research & Development on Severe Infectious Diseases, Kunming, Yunnan Province, People’s Republic of China; e Yunnan Key Laboratory of Vector-borne Infectious Disease, Kunming, Yunnan Province, People’s Republic of China

**Keywords:** Dengue virus, lipid metabolism, cholesterol, fatty acid, sphingolipid, phospholipid, antiviral, metabolic disorders

## Abstract

In recent years, Dengue virus (DENV) has continued to pose significant health risks in tropical and subtropical areas worldwide, raising health alerts worldwide. It can cause hyperviremia in humans and can even lead to fatal clinical diseases. The life cycle of DENV is intricately linked to cellular lipids, and the virus selectively utilizes relevant enzymes involved in lipid metabolism to modulate the existing metabolic system in host cells during entry, replication, assembly, and other stages, thereby creating an environment conducive to its complete replication cycle. At present, there is a lack of effective and specific anti-DENV treatment measures. This review summarizes the recently identified lipid metabolism molecules and metabolic related diseases that affect DENV infection, explores the dependence of DENV on lipid metabolism and provides potential targets for the treatment of dengue fever (DF).

## Epidemiology of dengue virus

Dengue virus (DENV), a member of the *Flavivirus* genus in the *Flaviviridae* family, is primarily transmitted through the blood-feeding activities of *Aedes aegypti* and *Aedes albopictus* mosquitoes. These mosquitoes serve as vectors, facilitating the transmission of the virus to humans each year, approximately 100–400 million people are infected with DENV, leading to around 500,000 severe cases and 20,000 fatalities due to hemorrhagic complications [1]. The global prevalence of dengue fever (DF) has surged in recent years, likely driven by factors such as global warming, urbanization, and population growth, all of which have contributed to the expansion of mosquito habitats [2]. Dengue transmission occurs when mosquitoes feed on viraemic hosts, transferring the virus to their midgut, where it replicates extensively. The virus then spreads to other tissues, including the lymph and salivary glands, which allows further transmission during subsequent mosquito feeding cycles [3]. The global incidence of DENV has risen with environmental and demographic changes, including warmer temperatures and increased urbanization. Major outbreaks are most common in tropical and subtropical regions, with peak incidences occurring every 3–5 years. DENV-1 and DENV-2 are the most prevalent serotypes, though DENV-3 and DENV-4 have also contributed to the global spread of the disease in recent years [4].

## Dengue virus life cycle in humans

DENV primarily infects monocytes, macrophages, and dendritic cells through receptor-mediated endocytosis. The acidic endosomal environment induces conformational changes in the envelope (E) protein, leading to membrane fusion and the release of the positive-sense single-stranded RNA (+ssRNA) genome into the cytoplasm. The viral RNA is directly translated into a polyprotein, which is cleaved into structural (capsid (C), precursor membrane (prM), E) and non-structural (NS1-NS5) proteins [5]. Replication occurs in endoplasmic reticulum (ER)-derived membrane compartments, where NS5 synthesizes a negative-strand RNA template for genomic RNA production. Newly synthesized RNA associates with C proteins to form nucleocapsids, which bud into ER and acquire an immature envelope containing prM-E proteins. These virions mature in the Golgi, where furin-mediated cleavage of prM produces infectious particles that are released via exocytosis. DENV evades immune detection by suppressing interferon (IFN) signalling through NS1, NS4B, and NS5 [6]. Additionally, antibody-dependent enhancement (ADE) facilitates viral entry into Fc receptor-bearing cells, increasing the risk of “severe dengue” manifestations such as dengue hemorrhagic fever (DHF) and dengue shock syndrome (DSS) [7].

## Challenges in dengue prevention and treatment

DENV infection is typically categorized into three phases: febrile, critical, and recovery. Most individuals experience mild or no symptoms, recovering within 1–2 weeks, but severe cases of DF can lead to DHF and DSS [8]. These severe forms of the disease present with symptoms such as abdominal pain, persistent vomiting, bleeding, and hypovolemic shock. Without timely intervention, DSS can result in fatal outcomes [9]. Currently, there is no antiviral treatment for dengue. The management of the disease relies on supportive care, including fluid replacement, antipyretics, and pain management. However, clinicians must carefully balance fluid therapy to avoid complications such as pulmonary edema. Diagnostic challenges further complicate dengue control efforts, particularly in resource-limited regions [10]. In these areas, logistical barriers – such as difficulties in sample collection, transportation, and testing – hinder routine DENV diagnostics. Additionally, the high proportion of asymptomatic cases (∼75% of infections) leads to underdiagnosis, misdiagnosis, or missed diagnoses, which impede outbreak response efforts [11]. The growing prevalence of DENV and its associated health burden highlight the need for better prevention and treatment strategies. Current dengue vaccine has shown to be effective in specific populations; however, it continues to face significant challenges, with ADE being a primary concern, followed by differential protection against different serotypes, and the associated complexity and cost of vaccination [12, 13]. These limitations emphasize the need for further research into novel approaches. Targeting lipid metabolism, a critical component in DENV infection, could offer a promising strategy for therapeutic interventions, as recent studies have highlighted the pivotal role of lipid pathways such as cholesterol, fatty acid (FA), sphingolipid (SP), and phospholipid (PL) in supporting viral replication and modulating immune responses during infection [14, 15]. By understanding how DENV manipulates host lipid pathways, we may identify potential drug targets that can disrupt the viral life cycle and reduce disease severity.

## Lipid metabolism in dengue virus infection

Lipid metabolism plays a fundamental role in the replication and survival of many viruses, including DENV. Lipid droplets (LDs), ER membranes, and cholesterol-rich microdomains are all essential components of the host cell machinery that DENV exploits. During infection, DENV induces changes in host lipid synthesis pathways, including the modulation of LDs, which provide a platform for viral replication [16]. Studies have demonstrated that lipid metabolism-related enzymes, such as acyl-CoA synthetase and phospholipases, are critical for the formation of viral replication complexes (VRCs) [17]. Furthermore, DENV induces the remodelling of the host cell membrane, which is essential for the assembly of viral particles. Lipids such as phosphoinositides (PtdIns) and SP are involved in the formation of membrane structures required for viral replication and budding [18]. These findings highlight the importance of lipid metabolism in the DENV life cycle and suggest that modulating lipid metabolism could provide a novel avenue for therapeutic intervention.

## Lipid metabolic pathways reprogrammed during DENV infection

### Fatty acid metabolism

FAs as constituents of triglyceride (TG), cholesterol esters, and PL, are categorized into saturated and unsaturated FAs, which can directly influence membrane curvature and fluidity. *De novo* lipid synthesis is upregulated in infected mosquito cells, primarily due to elevated levels of free fatty acids (FFAs) such as palmitic acid and stearic acid [19]. These are subsequently esterified to form TG, which are stored in LDs. Viruses can utilize these LDs to bind to the surface of C proteins, thereby promoting the formation of VRC. Additionally, these lipids can undergo β-oxidation in mitochondria to provide energy for viral replication. Heaton NS et al. [20] proposed that FA could be modified and incorporated into ER membranes in dengue-infected cells, or act synergistically with cholesterol, resulting in increased membrane fluidity. Villamor et al. [21] found that levels of docosahexaenoic acid (DHA) and arachidonic acid (AA) relative to total FAs were elevated in patients with severe dengue, while levels of dihomo-γ-linolenic acid (DGLA) and pentadecanoic acid were lower in patients with mild dengue. DHA is an omega-3 polyunsaturated fatty acid (PUFA) that plays a crucial role in regulating the production of inflammatory mediators and exerts anti-inflammatory effects. AA typically exists in cell membranes as PLs, which are released upon cell membrane stimulation during inflammatory responses, thereby triggering the inflammatory cascade. In a 2013 study, Cui et al. [22] observed that increased DHA levels in DF patients compared to healthy individuals could be an early anti-inflammatory response during the acute phase of infection, and that lower DHA levels were associated with more severe DF. Similarly, AA levels increased in the acute stage of infection and returned to normal during recovery. A 2017 study [23] utilizing a humanized mouse model of DENV infection revealed a significant increase in AA during the early stages of infection. Although the impact of elevated DHA on DENV infection remains unclear, studies suggest that DHA may have a protective role. DHA can competitively inhibit AA metabolism and reduce the production of pro-inflammatory mediators such as leukotrienes [24]. Additionally, DHA has been shown to decrease NF-κB signalling and reduce the expression of adhesion molecules on monocytes in endothelial cells, which could contribute to its anti-inflammatory effects. These findings may help explain the variations in disease severity observed among dengue patients. Further studies are needed to clarify DHA's precise role in modulating the immune response during DENV infection.

### Cholesterol metabolism

Cholesterol is a hydrophobic sterol molecule produced by cells and makes up approximately a quarter of the plasma membrane lipids. It helps regulate membrane fluidity, signal transduction, and transport of substances. Liver injury is the hallmark of DF patients, and its clinical manifestations are elevated levels of aspartate aminotransferase (AST) and alanine aminotransferase (ALT). Although the liver is the primary site of human cholesterol synthesis, dengue infection has been associated with an increased risk of liver steatosis, which leads to elevated lipid levels. Total cholesterol comprises low-density lipoprotein cholesterol (LDL-C), high-density lipoprotein cholesterol (HDL-C), and TG [25]. Significant reductions in serum total cholesterol levels, especially LDL-C, were found in patients with severe dengue disease. A study [26] utilizing confocal microscopy revealed an increased presence of low-density lipoprotein receptor (LDLR) on the plasma membrane of infected cells at 1 and 6 h post-infection (hpi). This study also demonstrated that LDL uptake was significantly higher in infected cells compared to uninfected cells. Furthermore, oxidized LDL (oxLDL) was shown to cross the vascular endothelial barrier and facilitate vascular penetration, suggesting a potential mechanism by which cholesterol metabolism may influence DENV pathogenesis [27]. Certain studies have demonstrated that the depletion of cholesterol through methyl-β-cyclodextrin reduces dengue infection, primarily in the intracellular replication phase and, to a lesser extent, during viral entry. Subsequently, the infection can be restored through cholesterol supplementation [28–30]. Moreover, after dengue infection in patients with hypocholesterolemia, the CD4^+^, CD8^+^ cell levels and related cytokines were significantly reduced. This suggests that low levels of cholesterol in the body may help fight viral infections.

### Phospholipid metabolism

PL consist mainly of plasma and endosomal membranes, including a hydrophilic head group, a glycerol skeleton and two hydrophobic fatty acyl chains. As the main structure of cell membrane, phosphatidylcholine (PC) and phosphatidylethanolamine (PE) accounted for more than 50% of cellular PL content. PL remodelling is accomplished through the Lands cycle, which increases the unsaturation of PL and promotes membrane fluidity. The Lands cycle is the deacylation of an acyl chain to form lysophospholipid (LPL), which is subsequently reacylated with a different acyl group to produce a new PL species. The *de novo* synthesis and remodelling of PL together ensure the maintenance and rearrangement of the membrane composition, which determines the properties and structure of the membrane. Metabolomic analysis showed that DENV actively inhibited the *de novo* PL synthesis pathway, but activated PL remodelling at denderated replication sites [31]. In the relevant research results [22, 23, 32], it was observed that the PC and lysophosphatidylcholine (LPC) changes in DF patients were lower than those in healthy people. Patients with DHF have higher PC levels than those with DF, and DHF is marked by high LPC, which rapidly promotes endothelial permeability. The level of PL in the midgut of *Aedes aegypti* infected with DENV [33] also showed a significant increase, which was consistent with the dynamics of viral replication in the midgut. When the virus replication reached the highest level in the midgut, the rapid increase in PL content led to a large expansion of the cell membrane to meet the assembly of the VRC.

### Sphingolipid metabolism

SP are FA that are amide-linked to a sphingoid base that bears a long-chain hydrocarbon, which include various forms of SP. They serve as ubiquitous component of eukaryotic cell membrane architecture and contribute significantly to the structural integrity of the cell membrane. *De novo* synthesis of SP occurs in the ER through a series of reactions to form ceramides (Cers), which form the central core of sphingomyelin (SM). An increase in Cer is often seen in DENV infection, possibly due to viral infection promoting the breakdown of SM [19, 34]. Changes in ceramide-precursor ceramide (Cer-DHCer) affect DENV genome replication and the release of infectious viruses. Moreover, SP may also be associated with membrane curvature, and the conical lipid structure of Cer induces spontaneous negative membrane curvature [35], which in turn affects the function of membrane microdomain, such as lipid rafts and vesicle transport, to affect cell signalling. In addition, Cer as a second messenger, can also induce apoptosis and autophagy, and induce apoptosis signal transduction and nitric oxide (NO) production through NF-κB under anti-DENV NS1 stimulation [35]. The newly synthesized Cer is converted by glycosyltransferase in the trans-Golgi into glycosphingolipids (GSLs), which bend and stretch the membrane and participate in the maturation and budding of the virus, a process that is also crucial for DENV replication [36].

### DENV-induced lipophagy and lipid droplets

When cells are under stress such as inadequate nutrition or severe injury, autophagy, an important defensive cellular programme, can maintain cell metabolic homeostasis and cell survival through intracellular degradation systems. Associated with DENV infection and lipid metabolism is a type of selective autophagy called lipophagy, in which autophagosomes can be called LD. LD is an important intracellular organelle primarily composed of TG and cholesterol esters, which are associated with intracellular lipid metabolism and substance transport. Studies have found that after DENV infection, lipid phagocytosis can mobilize lipids stored in host cells and enhance β-oxidation to provide energy for viral replication [37]. Simultaneously, LD can bind to viral proteins and RNA to provide a protective environment for viral. Interestingly, LD is also related to immune response, and the activation of Toll-like receptor (TLR) can induce the production of LD and promote the recruitment of signal molecules to the surface of LD, thus increasing the production of IFN [38]. Therefore, we speculate that LD may play a double-edged role in dengue infection, which can promote the replication of DENV and provide the host with antiviral immunity.

### Non-lytic release of dengue virus via exosomes

Exosomes are small vesicles formed and secreted by host cells through endocytosis, subsequently released extracellularly via multivesicular body (MVB) fusion with the plasma membrane. Unlike viruses that typically release progeny virus particles by lysing host cells, DENV can be released in a non-lytic manner via exosomes. The membrane structure of exosomes is primarily composed of lipid components from the host cell, and this lipid composition plays a crucial role in viral release and immune evasion. This mechanism not only minimizes damage to the host cell but also allows the virus to escape immune surveillance more effectively [39]. Exosomes derived from infected cells, such as macrophages, can interact with recipient cells through phosphatidylserine (PS) on their surface, facilitating viral uptake into these cells and supporting viral spread without triggering a strong immune response [40]. In addition, in recent studies by Li MY et al. [41], it was found that this non-lytic mode of viral release is generated in a virus-triggered SrC-family kinase (such as Lyn)-dependent manner and plays an important role in secretion of mature infectious viral progenies.

## Relationship between the life cycle of dengue virus and cellular lipids

**Figure 1.**
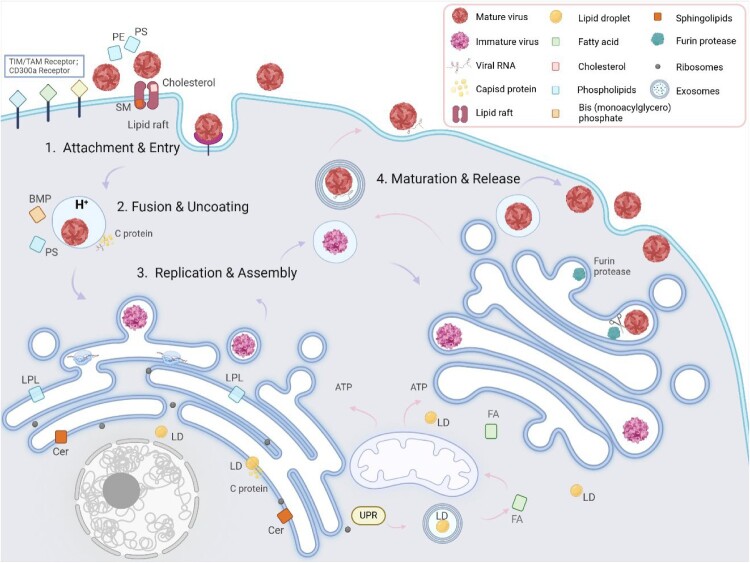
Involvement of related lipids in the DENV replication cycle: (1) Attachment & Entry: DENV binds to host receptors via PS and PE on its surface, interacting with TIM, TAM, and CD300a. Lipid rafts enriched in cholesterol and SM facilitate clathrin-mediated endocytosis. (2) Fusion & Uncoating: The acidic endosomal environment, along with anionic lipids such as BMP and PS, promotes membrane fusion and viral genome release. (3) Replication & Assembly: Viral replication occurs in ER-derived VRC, where Cer, LPL, FA, cholesterol, glycerophospholipids, and SP facilitate membrane remodelling. C proteins associate with LDs for nucleocapsid formation. DENV can also utilize secretory autophagy for non-lytic release, with autophagy-mediated LD degradation providing energy (pink arrow). (4) Maturation & Release: Immature virions undergo furin-mediated cleavage in the Golgi, forming mature particles that are released via exocytosis. This diagram was created using Biorender.com. PS, Phosphatidylserine; PE, Phosphatidylethanolamine; BMP, Bis (monacylglycerol) phosphate; TIM, T cell immunoglobulin mucin domain 1; TAM, Tyrosine protein kinase receptor family 3-AXL-MER; Cer, Ceramide; LD, Lipid droplet; ER, Endoplasmic reticulum; SM, Sphingomyelin; VRC, Viral replication complex; LPL, lysophospholipid; FA, Fatty acid; SP, Sphingolipid; UPR, Unfolded protein response; C, Capsid.

The results of lipid metabolomics and transcriptomics show that DENV infection reregulates lipid profiles such as PL and SP [31, 34, 42]. The initial step in the DENV life cycle involves adsorption to host cells. However, rather than entering cells through specific receptor binding mechanisms, dengue recognizes and attaches to a diverse array of molecules. Notably among these are T cell immunoglobulin mucin domain 1 (TIM) and members of Tyrosine protein kinase receptor family 3-AXL-MER (TAM), which serve as ligands for PS and PE present in the viral envelope [43]; Also, human CD300a can directly interact with them [44]. The second step is to introduce the virus into the cell through clathrin-mediated endocytosis, after the cell recognizes the virus. The acidic environment of the endosome triggers irreversible trimerization of the E protein so that the viral envelope fuses with the endosome membrane and release the viral RNA genome into the cytoplasm [45]. Certain lipids have the characteristics of polyunsaturation, charge difference, and structure, which can change membrane fluidity, physical and chemical properties, and promote membrane fusion. For instance, the incorporation of anionic lipids, including bis (monacylglycerol) phosphate (BMP) and PS, into the plasma membrane contributes to the induction of DENV plasma membrane fusion at low pH [46]. Lipid rafts, membrane microdomains enriched cholesterol and SP, promote conformational changes in the E protein and can also serve as a platform for viral transport to suitable intracellular sites (Figure 1). Lee et al. [47] showed that lipid rafts on the plasma membrane promote the entry of DENV, and the accumulation of cholesterol seemed to be effective in preventing viral fusion. However, an earlier report [48] showed that cholesterol was not necessary for entry of viral entry, so the role of cholesterol in this step needs to be further studied.

In the third step, following the entry of the virus into the host cell, cellular organelles are exploited for replication and assembly. Significant membrane reorganization occurred during this phase. The rough endoplasmic reticulum (RER) undergoes curvature to form an invaginated vesicle cluster (Ve) that houses VRCs, where viral RNA is translated and replicated. Smooth endoplasmic reticulum (SER) membranes develop convoluted membranes (CMs) at multiple sites for protein maturation. CMs can store lipids or interfere with innate immune responses by disrupting mitochondrial associated membranes (MAMs) or sequestrating innate immune pattern recognition receptors (PRRs). DENV assembles through genome replication and synthesis after interacting with C protein to establish a nucleocapsid complex while acquiring E protein and PrM protein from its environment [49]. The C protein is mediated by the protein on the surface of LD and binds to LD, providing a platform for assembly [50]. VRC requires FA, cholesterol, glycerophospholipids and SP dominated by Cer. Distinct lipid types facilitate viral replication through various mechanisms. For instance, LPL and Cer may induce membrane curvature, thereby promoting ER membrane remodelling. The process of the ER necessitates an increased supply of lipids, such as PC and PE, which are abundant in the ER menbrances [51]. The combination of Cer and LPC to induce negative and positive curvature, respectively, fulfils the requirements for ER membrane remodelling (Figure 1). Cholesterol and FA can supply energy necessary for viral replication through β-oxidation among other pathways. NS proteins are associated with lipid rafts within host cells' membranes, indicating that VRC may reside within these lipid raft structures [52]. Additionally, viral infection has been shown to disrupt ER homeostasis, leading to endoplasmic reticulum stress (ERS) responses characterized by an unfolded protein response (UPR), which subsequently initiates autophagy [53] (Figure 1). Autophagy plays a crucial role in membrane reorganization during DENV infection, as it involves multiple membranous events, including the conversion of LC3-I to LC3-II. Upon activation, double-membrane autophagosomes are formed, and autophagy-related proteins (Atgs) are expressed to regulate this process. LC3-I undergoes conjugation with PE, forming the lipidated LC3-II, which integrates into the autophagosomal membrane and is essential for autophagosome maturation. The co-localization of LC3 with dsRNA, DENV-NS1, and ribosomal protein L28 suggests that DENV replication is closely associated with autophagic vesicles [54]. Moreover, NS4A, a viral protein linked to autophagy, is a key component of the VRC. Its transmembrane domain is cleaved by host signalling enzymes within the ER lumen, and the mature NS4A facilitates membrane rearrangement by inducing ER membrane curvature, promoting phosphatidylinositol 3 kinase (PI3 K)-dependent autophagy, and enhancing cellular survival [55]. Finally, these newly synthesized immature virions are cleaved along the secretory pathway by the furin protease in the trans-Golgi apparatus to mature the PrM protein and release it outside the cell through exocytosis [56].

## Enzyme hijacking by DENV for lipid synthesis and metabolism

### Fatty acid metabolism-related enzymes in DENV infection

**Figure 2.**
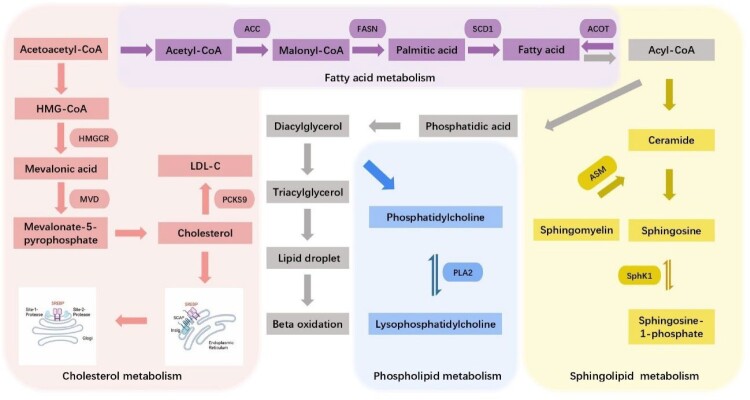
Enzymes associated with dengue virus infection in lipid metabolism: Different lipid metabolic pathways are classified according to different colours, with rectangular boxes representing metabolites and oval legends depicting enzymes acting on lipid metabolic pathways. To enhance the clarity of the legend, only selected key enzymes that have been shown to be associated with dengue virus infection (related to Table 1) are included, along with representative metabolites of these selected enzymes. HMGCR, 3-Hydroxy-3-Methylglutaryl-CoA Reductase; MVD, Mevalonate Diphosphate Decarboxylase; PCSK9, Proprotein Convertase Subtilisin/Kexin Type 9; LDL-C, Low-density lipoprotein cholesterol; ACC, Acetyl-CoA Carboxylase; FASN, Fatty Acid Synthase; SCD1, Stearoyl-CoA Desaturase-1; ACOT, Acyl-CoA thioeaterase; ASM, Acid Sphingomyelinase; SphK1, Sphingosine kinase 1; FA, Fatty acid; SP, Sphingolipid; PL, Phospholipid; PLA2, phospholipase A2-type enzymes.

DENV modulates lipid metabolism at different stages through related enzymes to facilitate lipid participation in the viral life cycle (Table 1), ensuring that it can fulfil essential functions in its life cycle (Figure 2). Fatty acid synthase (FASN) is a complete enzymatic system comprising two identical 272 kDa multifunctional peptides. DENV may interfere with FA synthesis by altering FASN localization. DENV NS3 is responsible for recruiting FASN to the VRC formation sites to produce FA. Then these can be modified and incorporated into ER membranes [20]. Rab18 may be involved in the interaction between FASN and DENV [57]. Collaboratively promoting membrane proliferation and rearrangement to facilitate relevant life-cycle steps, such as viral RNA replication and assembly. A key step in FA metabolism catalyzed by acetyl-CoA carboxylase (ACC) is the carboxylation of acetyl-CoA to produce malonyl-CoA, a rate-limiting substrate essential for *de novo* lipogenesis, and the inhibition of mitochondrial FA β-oxidation by inhibiting carnitine-palmitoyl transferase I (CPT-1) [58]. One study [44] has shown that following ACC inhibition, a variety of lipids are reduced and a large number of lipids required for degeneration, complex organization, and morphogenesis are consumed. It is hypothesized that ACC activity increases due to inactivation of AMPK during infection. Acyl-CoA thioeaterases (ACOTs) hydrolyze fatty acyl-CoA to FFAs and Coenzyme A. This process regulates lipid metabolism by maintaining the ratio between the two organelles. The function of ACOTs may be to reduce excessive β-oxidation by mediating the expulsion of FA from the mitochondrial matrix and indirectly inhibiting viral replication by modulating the expression of peroxisome ACOTs, thereby influencing peroxisome lipid degradation activity [59]. Stearoyl-CoA Desaturase-1 (SCD1) is a 40 kDa integrated membrane protein in ER. It regulates the balance between saturated and monounsaturated fatty acids (MUFAs) in cells. During the early stage of dengue infection, SCD1 increases MUFA production and enhances the fluidity of membrane contact sites, thereby facilitating viral replication, assembly and maturation by providing membrane contact sites to obtain requisite substrates and energy. In the later stages of infection, viral replication reaches maximal efficiency and excess FA negatively inhibits SCD1 production. Thus, SCD1 regulates DENV infection in a time-dependent manner [60]. These FA metabolism-related enzymes modulate membrane structure and lipid dynamics to support DENV infection. FASN and SCD1 drive membrane remodelling, while ACC and ACOTs regulate lipid synthesis and degradation. Their roles in membrane proliferation and curvature facilitate viral replication and assembly, highlighting potential antiviral targets in lipid pathways.

**Table 1.** Dengue virus completes their life cycle by hijacking the enzymes involved in lipid synthesis and metabolism.

Lipid class	Enzyme	Pathway of action	DENV infected	Phases that affect the virus	References
Fatty acids	Fatty acid synthase	*De novo* synthesis of fatty acids	Upregulated	Viral	[20, 57]
				Replication	
	Acetyl-CoA carboxylase	*De novo* fatty acid synthesis and fatty acid β-oxidation	Upregulated	Reduced viral replication and assembly	[58]
	Acyl-CoA thioeaterase	Fatty acid β-oxidation	Upregulated	Viral	[59]
				Replication	
	Stearoyl-CoA Desaturase-1	Desaturation of fatty acids in lipid metabolism	Upregulated	Viral	[60]
				Replication	
	3-hydroxy-3-methylglutaryl-CoA reductase	Key enzyme in cholesterol biosynthesis	Upregulated	Viral	[61]
				Replication; Morphogenesis; Assembly	
Cholesterol	PCSK9	Regulation of Cholesterol metabolism	Upregulated	An unidentified function in viral	[62]
				Replication	
	Mevalonate diphosphate decarboxylase	Key enzyme in cholesterol biosynthesis	Upregulated	Viral	[30]
				Replication	
Sphingolipids	Acid Sphingomyelinase	Ceramide synthesis and signalling	Upregulated	Affects multiple phases of viral life cycle	[63]
	Sphingosine Kinase 1	Regulation of sphingolipid metabolism	Upregulated	Innate immunity	[64]
Phospholipids	PLA2G4A	Production of arachidonic acid from membrane phospholipids	Upregulated	Morphogenesis; Assembly	[65]
	PLA2G7	Synthesize of Lysophosphatidylcholine	Upregulated	Virus fusion	[19]

### Cholesterol metabolism-related enzymes in DENV infection

3-hydroxy-3-methylglutaryl-CoA reductase (HMGCR) is a rate-limiting enzyme in cholesterol biosynthesis. The upstream molecules primarily involved in the phosphorylation of HMGCR at serine 872 are 5′ adenosine monophosphate (AMP)-AMPK and protein phosphatase 2A (PP2A); however, DENV infection increases HMGCR activity unregulated by PP2A. Therefore, reduced AMPK kinase activity prevents HMGCR phosphorylation, leading to an increase in its activity. The enhanced activity of HMGCR stimulates cholesterol accumulation in the ER. HMGCR can be observed in the VRC, where it is co-localized with cholesterol, and the viral proteins NS3, NS4A, and E. This shows that the enzyme was beneficial to the formation and structure maintenance of the VRC during this process [61]. Proprotein Convertase Subtilisin/Kexin Type 9 (PCSK9) is a negative regulator of LDLR. The elevation of PCSK9 under hypoxia conditions reduces results in a reduction of LDLR, which impacts the uptake of LDL-C and promotes *de novo* cholesterol synthesis. Relevant clinical data have shown a direct correlation between plasma levels of PCSK9 and higher levels of viraemia and disease severity in patients with dengue [62]. Mevalonate diphosphate decarboxylase (MVD) is an enzyme involved in the cholesterol biosynthesis pathway and promotes subgenomic replicons and live viral infections in dengue. Inhibition of this enzyme not only reduces the level of cellular cholesterol but also leads to an increase in phosphorylated intermediates related to the regulation of FASN. However, cholesterol cannot be utilized to rescue viral activity, suggesting that MVD may also be involved in pathways other than cholesterol metabolism that participate in the infection process [30]. These enzymes regulate cholesterol metabolism to support DENV infection. HMGCR enhances cholesterol accumulation for VRC formation, while PCSK9 reduces LDLR levels, influencing viral replication. MVD aids infection beyond cholesterol synthesis. Targeting these pathways may offer antiviral strategies.

### Sphingolipid pathway-related enzymes in DENV infection

Acid sphingomyelinase (ASM) is the most prevalent SP in eukaryotes. It catalyzes the decomposition of SM into Cer and phosphocholine. With the modulation of ASM, Cer can facilitate the release of new virions in infected host cells and regulate DENV-induced apoptosis of endothelial cells and immune evasion, which are conducive to viral infection [63]. Sphingosine kinase 1 (SphK) is a host cell enzyme that phosphorylates lipid sphingosine to form sphingosine-1-phosphate (S1P), which promotes DENV infection. Jennifer N. Clarke et al. [64] found that SphK1 might regulate innate immune responses influencing virus susceptibility to cells. SphK1 could be secondary to host cell responses, such as tumour necrosis factor α (TNF-α) production, and could promote early dengue infection. Overall, SP metabolism is critical in DENV infection. ASM aids virion release and immune evasion, while SphK1 promotes infection by regulating immune responses. Targeting these pathways could offer antiviral potential.

### Phospholipid pathway-related enzymes in DENV infection

More than 30 phospholipase A2-type enzymes (PLA2) encode enzymes involved in PL metabolism in the human genome, and Ca^2+^-dependent PLA2G4A specifically activates the release of AA. Subsequently, this process promotes the production of inflammatory mediators such as prostaglandins and leukocytes. In a study by Nicolas Menzel et al. [65], it was found that inhibition of PLA2G4A activity reduced the concentration of core proteins secreted by LDs, the core envelope, and particles. As AA and other PUFA increase membrane fluidity, varying levels of PLA2G4A may alter membrane properties. Additionally, LPC primarily results from the hydrolysis of PC by PLA2G7 [19]. LPC is an inverted conical structure that, if incorporated asymmetrically, results in positive curvature and induces vesicle fission and budding. Structural analysis of infected cell membranes revealed the presence of highly curved membranes and smaller vesicles [31]. Therefore, these enzymes in PL metabolism may alter the structure of these membranes by fission and budding vesicles and by enriching membrane curvature molecules to provide better assistance in dengue infection.

## Lipid metabolism as a therapeutic target for anti-DENV therapy

Arboviruses, such as DENV, pose a global public health challenge, underscoring the urgent need for specific antiviral therapeutics. However, vaccine development against dengue has been hampered by challenges such as ADE, high genetic variability of the virus, and other factors impacting vaccine safety and efficacy. Current treatments primarily address symptoms; however, pharmacokinetic issues and viral resistance often limit their effectiveness. Given the close relationship between DENV and host cell lipid metabolism, viral access to necessary substrates and energy-targeting lipid metabolic pathways represents a promising approach for antiviral therapy development (Table 2).

**Table 2.** Potential chemical compounds or drugs for therapeutics in DENV infections.

Lipid biogenesis process	Proposed target	Drug/inhibitor	Stage of action	*In vivo* mode of administration	Efficacy evaluation	Mechanism of action	References
Fatty acids Metabolism		C75; Cerulenin	Early infection	Injection	Reduced viral replication	Inhibition of FASN activity regulates fatty acid synthesis and reduces lipid droplet	[19, 20]
	FASN	Orlistat	Early infection	Oral	Reduced viral replication	Inhibition of FASN leads to a decrease in the ability of virus particles to form correctly and export from cells	[66]
	ACC	CP640186; PF-05175157; PF-05206574; PF-06256254; PF-06409577	Early infection	Oral	Destroy virus proliferation; Improve the survival rate of infected mice	Promotes ACC phosphorylation and regulate lipid droplet formation	[58, 67]
	SCD1	MK8245	Early infection	Oral	All four DENV serotypes showed antiviral activity	Regulation of UPR triggered by misfolding of viral glycoproteins in the ER	[68]
Cholesterol metabolism	HMG-CoA	Statins (Lovastatin; atorvastatin)	Early infection	Oral	Inhibit virus maturation and release; Prolonged mice survival	Reduces cholesterol synthesis through inhibition of HMGCR	[69–71]
	AMPK	Metformin; PF-06409577	Early infection	Oral	Variable effects across different cell types; Antiviral infection in low dose and low virulence	Inhibition of HMGCR activity by AMPK reduces cholesterol synthesis	[61, 72, 73]
	PCSK9	Alirocumab	Early infection	Injection	Decreased viremia, but could not be verified in mouse models	Inhibits cholesterol uptake by decreasing LDLr recycling	[74]
	SREBP	PF-429242; AEBSF	Early infection	Injection	Exerts suppressive effects on the viral replication of four DENV serotypes	The mechanism of the inhibitory effects is still unclear; Inhibition of HMGCR activity to reduce intracellular cholesterol levels	[75, 76]
	Desmosterol reductase; Oxidosqualene cyclase	U18666A	Early infection	Injection	Reduces viral replication	Prevents efficient viral fusion or the subsequent uncoating and suppresses *de novo* sterol biosynthesis	[77, 78]
Sphingolipid Metabolism	S1P	FTY720	Early infection	Injection	Restore endothelial barrier function	Activation of S1P reduces vasoactive mediators through sphingosine signalling	[79]
	Dihydroceramide Desaturase	4-HPR	Early infection	Oral	Provides Protection Against DENV Infection	Inhibits the interaction of the DENV NS5 protein with nuclear importin	[79]
Phospholipids Metabolism	Arachidonic Acid	Pyrrolidine-2	Early infection	Oral	Reduce DENV infectivity	Inhibition of PLA2G4A activity impairs infectivity of released particles	[65, 80]
	LPC	Darapladib	Early infection	Oral	Reduced viral replication	Inhibition of PLA2G7 affects membrane flow and impedes virus budding and assembly	[81]
Glycolysis	Lactate dehydrogenase; Hexokinase	Oxalate; 2DG	Early infection	Injection	Reduction in DENV replication	Inhibition of the glycolytic pathway	[82, 83]
Autophagy	PI3K	3-MA; Ka-003	Early infection	Injection	Suppresses DENV replication	Blocks the interaction between virus particles and autophagosomes	[37, 84, 85]

### Targeting fatty acid metabolism

The investigation of the FASN inhibitors C75 and Cerulenin revealed that their active sites in the β-ketoacyl synthase domain interact with cysteine, thereby inhibiting FA biosynthesis. Prompt treatment after DENV infection of mosquito cells can reduce viral replication [19] and diminish the number of LD in DENV-infected cells [20]. C75 exhibited a potent antiviral effect against dengue. It reveals no alteration in the entry or translation phase of the virus but demonstrates corresponding effects on replication and assembly. Orlistat, a drug that inhibits the FASN thioesterase domain, diminishes E protein expression in treated cells, thus indicating FASN's significance in the proper formation and subsequent export of new virions [66]. CP640186 is a potent inhibitor of mammalian ACC and exhibiting significant antiviral activity and low toxicity [67]. It does not prevent viral entry but inhibits dengue infection during the LD-consuming phases, thereby reducing the viral replication capacity and influencing assembly. Oral administration of CP640186 in a dengue-infected murine model demonstrated protective effects against mortality due to viral infections. Furthermore, PF-series compounds, such as PF-05175157, induce AMPK activation, resulting in the inhibitory phosphorylation of ACC and reduced DENV proliferation at non-cytotoxic concentrations [58]. The SCD1 inhibitor MK8245 is effective against all four serotypes. It damages the correct folding of NS1, E, PrM, and other viral proteins, inhibits viral replication, and promotes the recovery of cells from ERS, thereby exerting antiviral activity. If additional exogenous unsaturated FA were added in the presence of inhibitors, the DENV titres could be restored, indicating that MK8245 regulates the viral life cycle via the FA synthesis pathway [68]. However, in the AG129 mouse model, MK8245 did not prolong survival or attenuate viraemia.

### Targeting cholesterol metabolism

The need for cholesterol at different stages of DENV infection can also provide appropriate targets for anti-dengue drugs. Statins, such as lovastatin, atorvastatin, and other HMGCR inhibitors, can limit the synthesis or absorption of cholesterol within the human body. Notably, lovastatin treatment increased viral proteins and RNA following dengue infection, but decreased the release of infectious viral particles in the supernatant. It is hypothesized that the drug does not affect protein synthesis and RNA replication, but rather inhibits viral maturation and release [69]. In AG129 mouse models that permitted infection, treatment with lovastatin reduced inflammatory cell infiltration in hepatocytes, extended survival, and reduced viremia [86]. However, it has not shown good results in the clinical treatment of DF [70], which may be attributed to certain limitations in the study design, including delayed administration time and insufficient patient sample size. Furthermore, atorvastatin, like lovastatin, is classified as a lipophilic statin. AG129 mice infected with DENV were administered drug treatment, resulting in delayed weight loss and significantly increased survival. In contrast to lovastatin, atorvastatin exhibits higher bioavailability and a half-life of up to 14 h [71]. This characteristic allows for shorter treatment durations and more effective dosages, potentially reducing adverse effects. Metformin (Met) is commonly used to treat type 2 diabetes mellitus (DM). It increases AMPK, a cellular energy sensor, and suppresses HMGCR activity by regulating the transcription inhibitory steroid receptor coactivator 2 (SRC-2), thereby inhibiting cholesterol biosynthesis. Furthermore, AMPK influences FA metabolism, which explains why Met reduces viral particle levels more effectively than lovastatin. But studies [72] on the effects of Met on different cell lines have been inconsistent, possibly because Met mainly acts on the liver, which is the main organ infected by DENV. PF-06409577 [73], as another AMPK selective activator, showed antiviral activity in the μM range and may have better potential antiviral activity than Met in the mM range. Alirocumab, a monoclonal antibody, binds to and degrades free plasma PCSK9. Reduced PCSK9 activity causes the endosome to release LDL-C at low PH, and induces the LDLR conformation to facilitate recycling in the plasma membrane, thus diminishing cholesterol synthesis [74]. Subcutaneous administration of alirocumab attenuated the inflammatory response in infected cells and augmented antiviral responses such as IFN-β. However, the plasma cholesterol of mice is transported in high-density lipoprotein (HDL) independently of LDL, making it impossible to study the mechanism of action of PCSK9 in denting models [62]. Sterols Regulate Element-Binding Proteins (SREBPs), which act as transcription factors that bind to the ER and nuclear membrane, controlling the expression of key lipogenic enzymes and integrating multiple cellular signals to control lipogenesis and subsequent LD biogenesis. Recent studies have demonstrated that SREBPs are inactivated by binding to the SREBP cleavage-activating protein (Scap) and the ER-associated insulin inducer gene (Insig). When cholesterol levels in the ER are insufficient, SCAP transports SREBP to the Golgi network for cleavage and release by S1P and site-2 protease (S2P). Mature SREBP are subsequently translocated to the nucleus to activate the transcription of target genes essential for cholesterol and FA biosynthesis. PF-429242 [75] and serine protease inhibitor AEBSF [76] are S1P inhibitors that affect cellular cholesterol synthesis and inhibit the transmission of all DENV serotypes. Although PF-429242 appeared to reduce lipid levels, viral titres were not restored by additional lipid supplementation. It may interfere with viral replication by influencing the host cytokines involved in infection. AEBSF can inhibit mature viruses, but it has potential cytotoxicity, possibly reducing cholesterol levels in DENV-infected cells by inhibiting HMGCR activity. U18666A is a widely used chemical that has been reported to inhibit oxidosqualene cyclase and desmosterol reductase in the cholesterol biosynthesis pathway [77]. The inhibitor was found to prevent cholesterol from leaving late endosomes and lysosomes during early dengue infection, rendering the virus unable to carry out membrane fusion. Within 24 h of infection, a severe reduction in viral proteins was found [78]. At the same time, it also inhibits the level of the intermediate product of cholesterol biosynthesis, which reduces cholesterol in the ER compartment by approximately half and inhibits virus replication.

### Targeting sphingolipid metabolism

Enzyme inhibitors involved in SP metabolism can also be used as candidates against DENV infection. Fingolimod (FTY720), an FDA-approved immunomodulator for multiple sclerosis, acts as an agonist for S1P upon intracellular phosphorylation, thereby restoring endothelial permeability. Treatment with FTY720 fully restored the monolayer integrity of human microvascular endothelial cells infected with DENV. In AG129 mouse models of DENV infection, FTY720 treatment resulted in significant improvements in weight, with approximately 70% of the mice surviving until day 10, while all untreated DENV-infected animals succumbed. Additionally, FTY720 treatment led to the recovery of altered hemogram and liver function parameters, as well as a marked reduction in vascular leakage in A129 mice [87]. These findings suggest that modulation of the SP pathway via S1P may offer a promising therapeutic approach for DENV infection. 4-Hydroxyphenylretinamide (4-HPR) has been shown to increase dihydroceramide levels by stimulating *de novo* synthesis and inhibiting SM metabolism. It primarily disrupts the recognition of NS5 by the host nuclear import protein and activates the UPR in infected cells [88]. When 4-HPR was administered after DENV infection, a significant reduction in viral RNA levels was observed within 12 h, along with a delay in the appearance of detectable viral proteins. These results suggest that 4-HPR affects early stages of the infection cycle, including viral entry, translation, and genome replication, rather than virion assembly or release [89]. *In vivo*, 4-HPR treatment reduced inflammatory cytokine levels associated with dengue disease and extended the survival time of the infected mice compared to the control group [89]. These experimental findings underscore the potential therapeutic value of targeting SP metabolism and UPR activation in DENV infection [79].

### Targeting phospholipid metabolism

PC, PE, phosphatidylinositol (PI), PS, and SM in PL metabolism are the main cell membrane components. During DENV infection, the cell membrane undergoes drastic modifications, and the virus inhibits the cell cycle of the cell membrane by reconfiguring PL in humans and mosquitoes. AA, the cleavage product of PLA2G4A, serves as a precursor of bioactive lipid mediators and plays a crucial role in inflammatory processes. The researchers speculated that the inhibition of PLA2G4A by pyrrolidine-2 (Py-2) did not impede viral entry, but rather limited the core protein content in these organelles by reducing the biogenesis of LD [65]. Adding high doses of AA in the presence of Py-2 can partially restore the production of infectious DENV progeny, suggesting that PLA2G4A plays a specific role in viral infections [80]. In addition, darapladib, a LPC inhibitor, also decreased membrane fluidity. This disrupts viral emergence or interferes with components produced by the virus and the host for transport to viral assembly sites [81].

### Targeting other lipid-related pathways

The study [82] has shown that pyruvate derived from glucose can be converted into lactic acid under hypoxia, and then further react in mitochondria to synthesize acetyl-CoA. Subsequently, cytoplasmic acetyl-coenzyme A, a key substrate for lipid synthesis, is formed through the action of citrate enzymes. DENV replication may result in the depletion of key metabolites, thereby inducing an increase in glycolysis. Oxalate, a pyruvate analogue, can specifically inhibit lactate dehydrogenase. 2DG, a glucose analogue, inhibits hexokinase in the glycolytic pathway. Both compounds have been shown to reduce viral RNA levels post-treatment [83]. Autophagy is a critical cellular process that can be exploited in DENV infection. The autophagy inhibitor 3-methyladenine (3-MA) exerts its primary action by inhibiting type III PI3 K, thereby blocking autophagosome formation and limiting viral replication [84]. However, 3-MA exhibits a dual role: while it suppresses autophagy under starvation conditions, it can paradoxically enhance autophagic flux under normal physiological conditions [85]. To optimize the therapeutic efficacy of 3-MA against DENV, it is crucial to refine both the dosing regimen and the timing of administration. Additionally, the natural compound Ka-003 [37], derived from the mulberry plant, has been shown to reduce the number of autophagic vacuoles in a dose-dependent manner following autophagy induction, significantly decreasing DENV particle levels.

## Dengue virus infection and metabolic disease

Recent studies have identified metabolic disorders such as cardiovascular disease, diabetes, and obesity (Table 3) as indicators of increased susceptibility to DENV infection [90–94]. DM, an endothelial disease characterized by impaired glucose metabolism, can lead to vascular damage, diabetic ketoacidosis, fatty liver, dyslipidemia, hypertension, and cardiovascular complications if untreated. It [98] suggests DM is a significant co-factor in severe dengue symptoms. DM’s histopathological features include microvascular and macrovascular injuries, and disrupted insulin secretion affects NO and reactive oxygen species balance in endothelial cells, paralleling increased vascular permeability seen in DHF/ DSS. Blood glucose levels promote DENV infection in *Aedes aegypti* mosquitoes. Data analyzed by Ing-Kit Lee et al. [96] show dengue patients with optimal blood glucose levels have a lower risk of severe dengue, highlighting the importance of glucose control. Diabetic patients produce higher IL-4 and GM-CSF levels after dengue infection, and DENV infection in diabetic mice leads to stronger inflammation and prolonged viremia [99]. DHF/DSS correlates with DM and other diseases like asthma, allergies, hypertension, renal insufficiency, hyperlipidemia, and heart disease [95], all of which cause endothelial dysfunction, increased NO, cytokine storms, and vascular permeability, worsening dengue outcomes. Obesity, characterized by excessive fat deposition, is a risk factor for diabetes, hypertension, and cardiovascular disease, affecting metabolism, endocrine function, and immune response. Karine Beatriz Costa et al. [97] found that individuals with BMI ≥ 30 kg/m^2^ and a recent mild DF had higher body fat index (BFI), indicating a link between obesity and DENV infections. Obesity is associated with low-grade inflammation and pro-inflammatory cytokine production, leading to oxidative stress, atherosclerosis, and metabolic syndrome. Clinically, obese dengue patients exhibit severe symptoms such as dramatic platelet reduction, increased petechiae, elevated creatinine and liver enzyme levels, and prolonged hospital stays. Obese mice infected with DENV show increased inflammatory cytokine production, weight loss, and thrombocytopenia compared to healthy controls [100].

**Table 3.** Lipid metabolic comorbidities associated with dengue fever in recent years.

Metabolic related comorbidities	Country & Year of study	Sample size	Classifications of dengue severity	Diagnostic methods of DENV	References
Diabetes; Hypertension	From 2006 to 2008 in Singapore	818 DHF and 1467 DF patients	WHO 1997 criteria	Dengue Duo IgM & IgG Rapid Strip Test and positive dengue polymerase chain reaction (PCR) assay	[90]
Diabetes, Allergy, and Hypertension	Between 2003 and 2005 in Brazil	170 cases of DHF and 1175 controls	WHO 1997 criteria	Enzyme-linked immunosorbent assay (ELISA) for anti-dengue IgG antibodies	[95]
Diabetes; Hypertension	In Mysuru, Karnataka, India (January 2022-January 2023)	316 dengue cases	WHO case definition	Patients with positive for dengue NS1 antigen and IgM antibodies by enzyme-linked immunosorbent assay (MAC-ELISA)	[91]
Diabetes and Chronic Kidney Disease (CKD)	Between September and December 2015 in Kaohsiung, Taiwan	1655 DF patients	WHO 2009 guideline	ELISA for anti-dengue IgG antibodies	[96]
Hypertension; Diabetes; Chronic Obstructive Pulmonary Disease (COPD); CKD	Between January 2013 and December 2014 in Malaysia	322 fatal dengue cases	WHO 2009 guideline	ELISA for anti-dengue IgG and IgM antibodies, NS1 antigen; PCR	[92]
Obesity	Between September 2017 and June 2018 in Brazil	49 recent, inapparent dengue patients	None of the individuals were reactive for the NS1 antigen; then 29 were negative for DENV IgM and 20 were positive, suggesting recent inapparent dengue.	Patients with positive for DENV IgM antibodies by ELISA	[97]
Diabetes; Cardiac disorders; Asthma	From 1 January 2005 to 31 December 2008 in Singapore	1039 patients with dengue infection	WHO 1997 and 2009 dengue severity categories with available clinical	Dengue Duo IgM & IgG Rapid Strip Test and PCR	[93]
Obesity	In Malaysia from May 2016 to November 2017	335 patients with dengue infection	WHO 2009 guidelines	Dengue rapid combo test (NS1, IgM or IgG and IgM positive)	[94]

## Concluding remarks and outlook

Mosquito-borne viral infections, particularly DENV, are a global health challenge. The widespread circulation of all four DENV serotypes, along with mosquito transmission and favourable breeding conditions, heightens the risk of severe dengue. While DENV has not become a pandemic in all regions of China, the lack of targeted treatments and vaccines highlights the need for further research and innovative control strategies. Lipids are essential throughout the DENV life cycle, from entry into host cells to the release of new virions. This review summarizes lipid-related molecules and metabolic disorders impacting DENV infection and examines potential therapeutic compounds that target lipid metabolism to alleviate clinical symptoms. Key lipid metabolism regulators offer promising targets for DENV intervention. Future research should focus on understanding the role of lipids in DENV pathogenesis, particularly in viral replication and immune evasion. Challenges in drug development remain, including the need for effective therapies that target lipid metabolism and address viral mutation. By targeting lipid pathways, new therapeutic strategies could be developed to prevent and treat DENV infections, offering a significant advancement in antiviral therapies.
